# Clinical application of a fixed reference line in the ultrasound quantitative diagnosis of female pelvic organ prolapse

**DOI:** 10.1186/s12880-023-01013-6

**Published:** 2023-10-30

**Authors:** Xiaojuan Cao, Yuwen Qiu, Zhiyong Peng, Lan Chen, Li Zhou, Anwei Lu, Chunlin Chen, Ping Liu

**Affiliations:** 1 Department of Obstetrics and Gynecology, Nanfang Hospital , Southern Medical University, Guangzhou, Guangdong 510515 China; 2 Department of Obstetrics and Gynecology, Shenzhen Hospital, Southern Medical University, Shenzhen, Guangdong 518101 China; 3 Department of Ultrasound, Nanfang Hospital, Southern Medical University, Guangzhou, Guangdong 510515 China; 4 Department of Anesthesiology, Shenzhen Hospital, Southern Medical University, Shenzhen, Guangdong 518101 China

**Keywords:** Pelvic organ prolapse, Transperineal ultrasound, Pelvic organ prolapse quantification (POP-Q), Pelvic floor, Ultrasound

## Abstract

**Objective:**

This study explored using an improved ultrasound (US) for quantitative evaluation of the degree of pelvic organ prolapse(POP).

**Design:**

A transluminal probe was used to standardize ultrasound imaging of pelvic floor organ displacements. A US reference line was fixed between the lower edge of the pubic symphysis and the central axis of the pubic symphysis at a 30°counterclockwise angle.

**Method:**

Points Aa, Ba, C and Bp on pelvic organ prolapse quantification (POP-Q) were then compared with the points on pelvic floor ultrasound (PFUS).

**Results:**

One hundred thirteen patients were included in the analysis of the standard US plane. Correlations were good in the anterior and middle compartments (PBN:Aa, ICC = 0.922; PBB:Ba, ICC = 0.923; and PC:C, ICC = 0.925), and Bland-Altman statistical maps corresponding to the average difference around the 30°horizontal line were close to 0. Correlations were poor in the posterior compartment (PRA:Bp, ICC = 0.444). However, eight (7.1%) cases of intestinal hernia and 21 (18.6%) cases of rectocele were diagnosed.

**Conclusions:**

Introital PFUS using an intracavitary probe, which is gently placed at the introitus of the urethra and the vagina, may be accurately used to evaluate organ displacement. The application of a 30°horizontal line may improve the repeatability of the US diagnosis of POP.

## Introduction

Pelvic organ prolapse (POP) is defined as descent of the pelvic organs and the adjacent vaginal wall from the normal position to the vagina or prolapse from the vagina [1]. The condition is present in more than 40% of women aged 50–79 years, and whom suffer from varying degrees of POP [2]. The clinical management of POP relies on patient symptoms and determination of the level of prolapse by physical examination to guide surgical plans and therapeutic strategies. The POP quantification (POP-Q) system, requires extensive knowledge in order to understand how the nine measured points are derived [3]. The POP-Q system is now accepted as the standard classification system for describing pelvic organ support defects [4]. The POP-Q is a relatively complex system, there is less acceptance of the system outside the field of urogynecology, Auwad et al. studied the results reveal that only 40.2% of the respondents routinely use the POPQ in their clinical practice and 33.5% never use it [5]. Among the urogynecologic subspecialists, 70% used the POP-Q system in their practice [6].

Although the POP-Q system provides a means of communicating the details of pelvic support deficiencies, it does not provide a clear definition of POP based on anatomical landmarks [7]. Therefore, appropriate auxiliary examinations may be a beneficial supplement to POP-Q assessment.

Pelvic floor ultrasound (PFUS) is a low-cost, radiation-free, real-time assessment for evaluating pelvic floor anatomy or function [8]. A standardization practice parameter of PFUS developed by IUGA - the horizontal line (H-line), which is defined as a horizontal reference line that passes the inferior-most point of the symphysis pubisis, is recommended in order to measure the descending distance of the pelvic organs [9]. Dietz et al. [10] found that US measurements using the H-line were almost linearly associated with POP-Q coordinates. However, in a study by Lone [11] et al. they demonstrated that the proportion of correct assessments was only 59.6%, 61.5% and 32.6% for bladder, bowel and middle compartment prolapse, respectively, when using the US H-line to evaluate patients with prolapse inside the hymen. Magnetic resonance imaging (MRI) can be used to establish reference lines according to different bone markers for use in different evaluation systems, such as the sacrococcygeal-inferior pubic point (SCIPP) line, horizontal line (H-line), pubococcygeal line (PCL), perineal line, and midpubic line (MPL) [12, 13]. However, MRI-based quantification methods tend to have poor consistency when compared with the clinical POP-Q scoring system, along with high cost, limited availability, and poor dynamic imaging that ultimately limit their widespread use. Najjari et al. [14] compared all US data based on the MPL (which refers to the line drawn through the central point and parallel to the longitudinal axis of the symphysis pubic bone) and H-line with the outcome of POP-Q and determined that the distance between the lowest point of the bladder at rest and Valsalva changed when using the horizontal line; however, the distance was constant when using the MPL. Volloyhaug [15] et al. similarly reported a higher misdiagnosis rate for TPUS when compared toPOP-Q. The variations in the defined reference lines thus result in significantly reducedvalues of TPUS [16]. This suggests that the accuracy of pelvic floor US staging may be limited by the US H-line. Therefore, to improve the repeatability of ultrasound inspection, it may be beneficial to set a fixed reference line.

## Materials and methods

This prospective observational study was reviewed and approved by the Medical Ethics Committee (NFEC2016166) and was performed by clinical and US diagnosticians using a double-blind approach. POP-Q was performed by a single urogynecologist specialist who had over 30 years’ worth of clinical experience at the time of the study. The inclusion criteria were as follows: a diagnosis of POP stage 0-IV; instruction in performing pelvic floor muscle contraction and the Valsalva maneuver; and POP-Q and introital PFUS conducted at the same time. The exclusion criteria were urinary lower reproductive tract and digestive tract tumors, pelvic floor spasm syndrome, ineffective Valsalva maneuvers, and inability to obtain standard introital PFUS planes. Finally, a total of 113 consecutive patients were enrolled between March 2017 and March 2019.

This study used US instruments and models that included the US GE Voluson E8 US system and an RIC5-9-D intracavitary probe (5–9 MHz; GE Medical Systems, Zipf, Austria) in pelvic floor US mode for real-time two-dimensional (2D) and three-dimensional (3D) imaging. US was performed by the same senior sonographer for all patients.

**Fig. 1:**
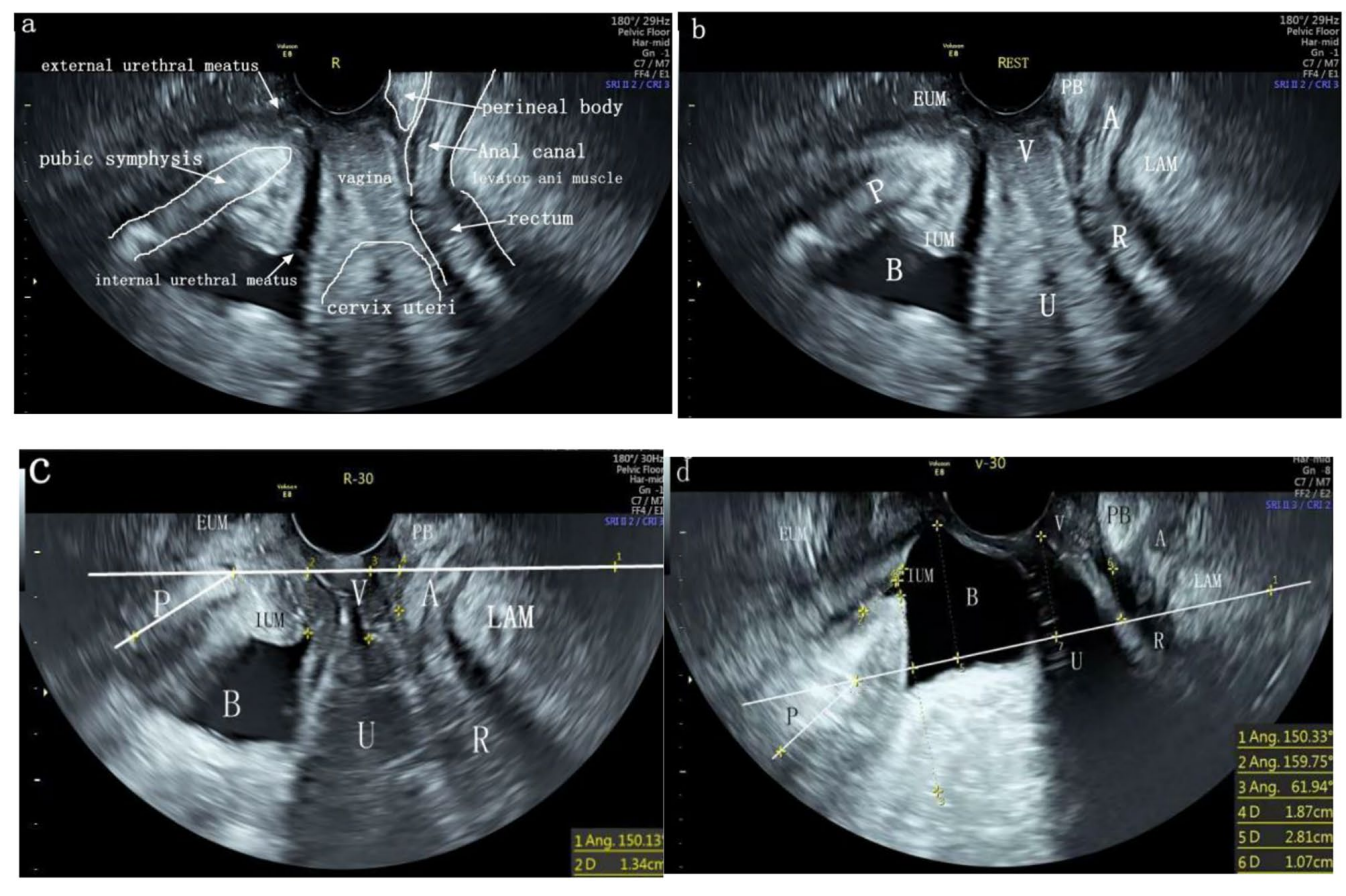
Images of the standard ultrasound plane of the pelvic floor organ. The distances between the organs and the 30° reference line of the ultrasound were measured when the women were at rest and exerting the maximum Valsalva breath-holding force (c and d). Pubic symphysis (P), bladder (B), vagina (V), cervix uteri (U), rectum (R), anal canal (A), perineal body (PB), internal urethral meatus (IUM), external urethral meatus (EUM), levator ani muscle (LAM)

Prior to each measurement, each female patient emptied their bladder and assumed the bladder lithotomy position (an enema was used to induce defecation when rectal stool interfered with imaging). The probe was then gently placed at the introitus of the urethra and the vagina. Static and real-time dynamic 2D and 3D sonograms of pelvic tissues and organs were retained for evaluation. During performance of the Valsalva maneuver, each patient held the sides of the examination bed with both hands in order to simulate the same force as the maximum strength at delivery and to ensure that the probe did not move with the organ. For each patient a total of three images were captured. Once the pictures were obtained, two observers blindly measured the distance between the 30° horizontal reference line and the static and maximum Valsalva force of the pelvic organs. Quality control ultrasound images was undertaken prior to measurement. Standard US section requirements were adopted in this study that included: The full picture of the median sagittal section ofthe pubic symphysis, urethra, bladder,vagina, cervix,uterus (if possible), anorectum, and perineal body were all clearly displayed simultaneously (Fig. 1a). The pubic symphysis typically had a slightly higher echo with a flat, oval shape (Fig. 1).The key point of quality control was identified to occur with the median sagittal plane of the pubic symphysis, and internal and external orifices of the urethra, anorectum, and perineal body all displayed on the ultrasound plane.Any missing datapoint indicates a nonstandard ultrasoundmedian sagittal plane.

The 30°H-line is a horizontal reference line that takes the MPL as the central axis, passes through the lowest point of the pubic symphysis, and rotates 30° counterclockwise. The ultrasound observation points when the pelvic organs descend are as follows: PBN refers tothe position of the apex of the posterior angle of the bladder and urethra. PBB refers tothe position of the lowest point after the bottom of the bladder descends. PC refers tothe position of the lowest point after the cervix descends. After hysterectomy, this is the lowest point after the vaginal stump descends. PRA means: The position of the lowest point after the anterior wall of the rectal ampulla descends. The distances between the PBN, PBB, PC, PRA and the 30° H-line were measured when the woman was at rest and under the maximum Valsalva force (Fig. 1). The measured values are reported in mm, measurements below the 30° H-line are positive, above are negative and 0 on the reference line. (Fig. 1).

In the absence of a reference standard for quantitative US indexing, a reference standard for indexing was established based on POP-Q evaluation features, MRI and the preliminary results of the introital PFUS in this study. The US indexing criteria were quantified using measurements collected under the maximum Valsalva force. A stage 0 result indicated that the distal end of the prolapse organ was located > 10 mm from the reference line on the side closer to the head (i.e., the quantitative value was <-10 mm). A stage I result indicated that the distal end of the prolapse organ was located within 10 mm of the reference line on the side closer to the head (i.e., the quantitative value ranged from >-10 mm to < 0 mm). A stage II result indicated that the distal horizontal reference line of the prolapse was located within 20 mm of the reference line on the side closer to the foot (i.e., the quantitative value ranged from ≥ 0 mm to ≤ 20 mm). A stage III result indicated that the protrusion was located at the farthest end of the reference line and was located more than 20 mm under the reference line on the side that was closer to the foot (i.e., the quantitative value was greater than 20 mm), but the apexes of the prolapsed organs were not completely below the reference line. A stage IV result indicated that the apexes of the prolapsed organs were all below the reference line. Perineal hypermobility was determined by referring to previously reported qualitative diagnostic criteria [17].

### Statistical analysis

All statistical analyses were performed using IBM SPSS 20.0 statistical analysis software. A two-observer agreement was tested using the intraclass correlation coefficient (ICC) two-way stochastic, absolute agreement model. US and POP-Q measurements along with grading (the PBN points correspond to the Aa points, the PBB points correspond to the Ba points, the PC points correspond to C points, and the PRA points correspond to the Bp points) were then statistically analyzed. All measurement data are presented as the mean ± standard deviation. Nonparametric tests were used to analyze the quantitative distal measurements. The Wilcoxon signed-rank test was used to compare paired samples, with P < 0.05 considered statistically significant. Analysis of the consistency between the US measurements and POP-Q measurements was performed by calculating the intraclass correlation coefficients (ICCs). Bland-Altman plots were also created.In the Mann-Whitney Test of rank sum test of graded data, P < 0.05 is considered to be the method of US and POP-Q measurement scale for ultrasound and clinical POP-Q measurement scale. Statistical differences of P > 0.05 were considered to indicate no statistical difference between the two scale methods. The agreement between US measurements and POP-Q measurements was analyzed by calculating intraclass correlation coefficients (ICCs). 0 was defined as unreliable, 1 as completely credible, less than 0.4 as poor consistency, greater than 0.75 as good consistency, and 0.75 ≥ ICC ≥ 0.4 as medium consistency. Draw Bland-Altman statistical charts and make tables, charts showing POP-Q assessment (Aa, Ba, C, Bp) and ultrasound assessment of pelvic urethral orifice, bladder, uterus and rectal ampulla (PBN, PBB, PC, PRA) were employed withmeasured values and relationships in staging. Calculations of the correlation coefficient (r), with P < 0.05 indicating statistical significance.

## Results

From a total of 165 consecutive patients, 52 were excluded from the study (urinary tumor in five, cervical tumor in six, pelvic floor spasm syndrome in eleven, ineffective Valsalva maneuver in twenty and failure to obtain a standard US plane in ten); 113 patients (hysterectomy in thirty-one) had a standard US plane of the pelvic floor organs and effective Valsalva maneuvers and were included in the final analysis. The image acquisition rate of the standard US plane of the pelvic floor organs using a transluminal probe was 93.9%. The general clinical and ultrasonographic data are presented in Table 1.The intraclass correlation coefficient (ICC) of the values measured by the two ultrasound observers fluctuated between 0.787 and 0.913. The consistency of the values measured by the two observers was high and the repeatability was good. The maximum value measured in the Valsalva state and the POP-Q value were taken and used for statistical analysis.

**Table 1:** General data of 113 female patients with pelvic floor dysfunction

Normal information	Range (maximum and minimum)	Mean ± the standard deviation
Age (years)	26 ~ 85	56.35 ± 12.94
Body weight (kg)	40–94	56.62 ± 8.75
Height (cm)	140–170	156.7 ± 5.08
Body mass index (kg/m2)	17.22 ~ 35.82	23.03 ± 3.14
Pregnancy (number)	0 ~ 13	3.55 ± 2.06
Births (number)	0 ~ 9	2.63 ± 1.48
Ultrasound measurement of the cervical length (mm)	21 ~ 78	38.57 ± 11.26
Ultrasound estimation of the bladder residual urine volume (ml)	0 ~ 96	27.92 ± 23.71
Ultrasound measurement of bladder wall thickness (mm)	3.0 ~ 7.8	4.83 ± 0.95
Anal levator muscle area at rest (cm2)	10.25 ~ 36.84	23.65 ± 5.1
Anal levator muscle area when tightening the anus (cm2)	9.74 ~ 30.44	20.42 ± 4.69
Valsalva pulling of the area of the levator ani muscle when the breath is strong (cm2)	15.73 ~ 47.56	30.79 ± 7.35

**Fig. 2:**
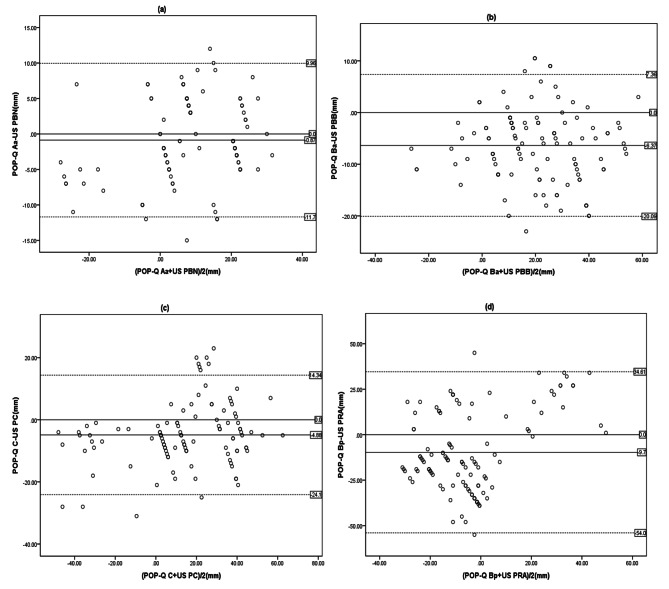
Bland-Altman scatter plots of the differences between the ITUS measurements and POP-Q measurements. The dotted lines represent the mean ± 1.96 SD (95% confidence interval) of differences, the solid line represents zero and the mean. Each dot represents one woman. The plots show narrower limits of agreements for PBN:Aa estimates. The differences between the PBN, PBB, and PC measurements and the Aa, Ba, and C values fluctuated above and below the average difference line; the average difference was close to 0

All data revealed a nonnormal distribution and uneven variance. No significant differences were observed between the US PBN value and the POP-Q Aa value (P = 0.285). However, significant differences were observed between the other values (P = 0.0001; Table 2). POP-Q was measured in centimeters after being clinically quantified and converted to millimeters for statistical analysis. As shown in Table 3, the detection ratio of prolapse graded POP-Q stage II and above was determined at different anatomical indication points, namely, Aa (101/113; 89.4%), Ba (104/113; 92.0%), C (94/113; 83.2%), and Bp (38/113; 33.6%). The P values of the PBN, PBB, and PC values and the POP-Q Aa, Ba, and C values were all P > 0.05, and no significant differences were observed between these values (Table 4). The values of PRA and Bp were calculated to be P < 0.05, indicating significant differences (Table 4). The correlations between the PBN, PBB, PC, and PRA values and the POP-Q Aa, Ba, C, and Bp values were analyzed by calculating the ICCs. The consistency of the PBN, PBB, and PC values with the Aa, Ba and C values was good; however, the consistency of the PRA value with the Bp value was poor (Table 5). Application of the diagnostic criteria [17] and the 30°reference line identified 8 (7.1%) cases of intestinal hernia (above the hymen in three), 37 (32.7%) cases of perineal hypermobility, and 21 (18.6%) cases of rectocele. Intestinal hernia and rectocele diagnosed by US were all clearly diagnosed during POP surgery. In Fig. 2, the Bland-Altman scatter plots show narrower limits of agreement for the PBN:Aa estimates. The differences between the PBN, PBB, and PC measurements and the Aa, Ba, and C values fluctuated above and below the average difference line; the average difference was close to 0.

**Table 2:** The Aa, Ba, C and Bp values of POP-Q were matched with the PBN, PBB, PC and PRA values of PFUS at maximum Valsalva for Wilcoxon symbol rank test

Measurement site		Mean ± standard deviation		Z	p
PFUS	POP-Q	PFUS (mm)	POP-Q(mm)		
PBN	Aa	9.46 ± 13.37	8.60 ± 14.56	-1.309	0.191
PBB	Ba	23.57 ± 18.05	17.20 ± 17.71	-7.386	0.000
PC	C	16.56 ± 24.05	11.68 ± 26.43	-5.283	0.000
PRA	Bp	0.85 ± 17.41	-8.85 ± 24.82	-4.156	0.000

**Table 3:** The different stages between the POP-Q quantitative value (Aa, Ba, C, Bp) using the hymen reference line and the PFUS quantitative value (PBN, PBB, PC, PRA) using the 30° reference line, the number and proportion of cases are shown in the table below

stage	PBN	Aa	PBB	Ba	PC	C	PRA	Bp
Stage 0	4 (3.5)	6(5.3)	3(2.7)	3(2.7)	16(14.2)	14 (12.4)	33(29.2)	26(23.0)
Stage I	8(7.1)	6(5.3)	9(8.0)	6(5.3)	2(1.8)	4(4.4)	17(15.0)	49(43.4)
Stage II	58 (51.3)	55(48.7)	45(39.8)	40(35.4)	51(45.1)	42(37.2)	55(48.7)	19(16.8)
Stage III	40 (35.4)	44(38.9)	49(43.4)	57(50.4)	41(36.3)	49(43.4)	8(7.1)	16(14.2)
Stage IV	3 (2.7)	2(1.8)	7(6.2)	7(6.2)	3(2.7)	3(2.7)	0(0)	3(2.7)

**Table 4:** “Mann-Whitney U” rank sum test of the Aa, Ba, C and Bp (POP-Q) Stages and the PBN, PBB, PC, and PRA (PFUS) grades

Measurement site		Rank mean (rank sum)		Z	p	Mann-Whitney U	Wilcoxon W
PFUS	POP-Q	PFUS	POP-Q				
PBN	Aa	121.07(13681.0)	105.93(11970.0)	-1.781	0.075	5529.0	11970.0
PBB	Ba	105.67(11941.0)	121.33(11941.0)	-1.837	0.066	5500.0	11941.0
PC	C	119.14(13462.5)	107.86(12188.5)	-1.319	0.187	817.5	12188.5
PRA	Bp	133.76(15115.0)	93.24(10536.0)	-4.731	0.000	4095.0	10536.0

**Table 5:** Consistency (ICC) analysis results of PBN, PBB,PC and PRAmeasured by PFUS with Aa, Ba,C andBp measured by POP-Q

Measurement site		Correlation coefficient	p	95% confidence interval	
PFUS	POP-Q			Lower limit	Upper limit
PBN	Aa	0.922	0.000	0.889	0.945
PBB	Ba	0.923	0.000	0.891	0.947
PC	C	0.925	0.000	0.893	0.947
PRA	Bp	0.444	0.000	0.283	0.581

## Discussion

### The use of intracavitary probes placed at the urethra and vaginal opening can better quantitatively evaluate patients with prolapse of POP-Q stage II and above

Transperineum PFUS, which places an abdominal transducer on the perineum/vulva for examination, has been used clinically for over 20 years. However, the technique often suffers from inconsistent US results due to the Variable reference lines and a non-standard ultrasound imaging plane [10, 11, 13–16, 18, 19]. Recently, high-resolution 3D transvaginal US technology has been used to improve tissue resolution. However, the anatomy and movement of the pelvic floor organs can beimpeded by the presence of the transducer in the vagina [20, 21]. In this study, an RIC5-9-D intracavitary probe was used, which allows obtaining high-resolution 3D transvaginal images.As shown in Fig. 1, the use of intracavity ultrasound probes placed at the urethral and vaginal openings can accurately locate the specific descending anatomical sites for imaging the urethra, bladder, uterus, and rectal ampulla. Among them,, the prolapse of POP-Q stage II and above accounted for a high proportion, such as Aa (101/113; 89.4%), Ba (104/113; 92.0%), and C (94/113; 83.2%) (see Table 3). When combined with the results shown in Table 4 the following could be determined: In comparison of ultrasound 30°horizontal reference line measurement value staging, the staging data of ultrasound measurement values (PBN, PBB, PC) and POP-Q measurement values (Aa, Ba, C) no statistically differences between the two staging methods in the evaluation of the anterior and middle chambers were identified; therefore there is little difference in the clinical application of the two staging methods. Combining the ICC correlation coefficients of the two methods, the anterior and middle compartments were well correlated (PBN:Aa, ICC = 0.922; Ba, ICC = 0.923; PC:C, ICC = 0.925). Based on theseresults, we concluded that the use of intraluminal high-resolution 3D ultrasound probes placed at the urethra and vaginal introitus can be used to evaluate patients better quantitatively with POP-QII stage and above prolapse of the anterior and middle compartments. Based on clinical experience, we suggest that the possible reasons for this finding are as follows: the resolution of the intracavity probe is high, and the contact surface of the intracavity probe is small, which does not affect the downward movement of prolapsed organs. When the maximum Valsalva breath force is reached, the probe takes the pubic symphysis as the fulcrum and is not easily pushed away by the prolapsed organ, thereby avoiding measurement errors after the probe moves. However, when the distal end of the severely prolapsed organ was found to exceed the imaging range of the intraluminal probe, the proximal end of the prolapsed organ couldbe visualized in the US plane. This provides a truer measurement of the distance between the proximal end and the fixed reference line to assess severe prolapse.

### Compared with the outcome of POP-Q, the 30°reference line has high consistency in the assessment of POP in anterior and middle chambers

DeLancey J. [22] proposed that the ideal evaluation system requires a fixed reference line and a true evaluation of organ displacement. Lines on US images, such as the H-line, MPL and PCL, were comprehensively analyzed by Gao et al. [23], however, no conclusion was reached on which reference line was best. Dietz et al. [10] found that US measurements using the H-line were associated almost linearly with POP-Q coordinates. Najjari et al. [14] measured the distances between the furthest descending point of the bladder and the MPL and H-line. The distance was constant when using the MPL. Taking the MPL may reduce operator bias to some extent; however, it is not parallel to the hymen. In clinical examination, the POP-Q system [3] quantifies the degree of organ prolapse by measuring the distance between the hymen and the indicator point in the vagina. Based on the above research combined with our clinical practice, we choose to use the 30°H-line, a horizontal reference line that takes the MPL as the central axis, passes through the lowest point of the pubic symphysis, and rotates 30°counterclockwise. Results from this study showed that this line is a fixed ultrasound reference line similar to that of the leading edge of the hymen, which is normally used to quantify the degree of POP. All US data were compared with the outcome of POP-Q, with the finding that correlations were good in the anterior and middle compartments, and Bland-Altman plots corresponding to the average difference around the 30° reference line showed values close to 0, indicating that the fixed US reference line has high consistency in the assessment of POP in anterior and middle chambers. Because the 30° H-line was fixed by the included angle, it was not affected by movement of the pelvic floor anatomy or organs, and thus was not affected by the pelvic tilt angle. Determining whether the 30°H-line on US can be better aligned with the physiological curvature of the human pelvis and made more similar to the horizontal reference line of the human body requires further research.

### The introital PFUS with the 30°H-line maybe a better method to quantify intestinal hernias

Regarding posterior pelvic assessment, although the reference line on the median pelvic organs was standardized in the evaluation of the posterior pelvic rectum, the consistency of the rectal ampullary decline between US and POP-Q was extremely poor (ICC = 0.444)and lead to overdiagnosis of rectal ampulla prolapse. Due to the particularity of the anatomical mechanics of the anterior and posterior walls of the rectal ampulla and the diversity of the descending direction, US images cannot currently be used to determine the demarcation point between the rectum and the colon. Therefore, the value of this PFUS assessment system to assess rectal ampulla decline is limited. However, in the eight cases of intestinal hernia and above the hymen in three patients diagnosed by US were all clearly diagnosed during POP surgery. This shows that the assessment system of introital PFUS with the 30°H-line maybe a better method to quantify intestinal hernias.

## Conclusions

Introital PFUS with an intracavitary probe, which is gently placed at the introitus of the urethra and the vaginamay accurately evaluate organ displacement. Compared with the outcome of POP-Q, the 30°reference line has high consistency in the assessment of POP in anterior and middle chambers, and maybe was a better method to quantify intestinal hernias. The application of a 30°horizontal line may improve the repeatability of the US diagnosis of POP.

## Data Availability

The data that support the findings of this study are available from Nanfang Hospital, Southern Medical University, but restrictions apply to the availability of these data, which were used under license for the current study, and so are not publicly available. Data are however available from the authors upon reasonable request and with permission of Nanfang Hospital, Southern Medical University. Some datasets generated and/or analysed during the current study are available in the [Clinical research integration platform software] repository, [PERSISTENT WEB (https://rdr.smuszh.com/fm/Display?id=3053)LINK TO DATASETS].
